# The Antimicrobial Efficacy of Sodium Hypochlorite and Chlorhexidine in Gutta-Percha Cone Decontamination: A Systematic Review

**DOI:** 10.3390/ma18071539

**Published:** 2025-03-28

**Authors:** Ruta Aucinaite, Egle Nedzinskiene, Vytaute Peciuliene, Irma Dumbryte

**Affiliations:** Institute of Dentistry, Faculty of Medicine, Vilnius University, Zalgirio 115, 08217 Vilnius, Lithuania; vytaute.peciuliene@mf.vu.lt (V.P.); irma.dumbryte@mf.vu.lt (I.D.)

**Keywords:** disinfecting solutions, irrigants, antibacterial, cross-contamination, *Enterococcus faecalis*, *Staphylococcus aureus*, *Candida albicans*, obturation materials

## Abstract

This systematic review aims to compare the efficacy of sodium hypochlorite (NaOCl) and chlorhexidine digluconate (CHX) in decontaminating gutta-percha (GP) cones against endodontic pathogens—*Enterococcus faecalis* (*E. faecalis*), *Staphylococcus aureus* (*S. aureus*), and *Candida albicans* (*C. albicans*)—within 0–10 min. A systematic search was conducted in six databases (PubMed, Web of Science, Cochrane Library, SCIELO, Scopus, and LILACS), supplemented by manual searches performed independently by three reviewers. No publication year restrictions were applied, and only English-language studies were included. This review followed the PRISMA statement (Preferred Reporting Items for Systematic Reviews and Meta-Analyses). The risk of bias was assessed using six parameters with a modified Cochrane risk of bias tool. Out of 309 potentially eligible studies, 216 were screened by title and abstract, 32 were selected for full-text assessments, and 7 were included. All studies had a moderate or high risk of bias. The majority of the included studies showed that higher NaOCl concentrations effectively eliminate *E. faecalis* and *S. aureus* within 1–5 min. However, data on CHX’s antimicrobial effect on *C. albicans* were limited. The qualitative analysis suggests that NaOCl remains the most effective agent for GP decontamination, while CHX with additives shows potential against fungal species.

## 1. Introduction

The success and longevity of endodontic treatment rely on eliminating bacteria from the root canal system, which can be achieved through effective chemomechanical debridement, proper root canal filling, and adequate restoration [1,2]. Endodontic treatment focuses on eradicating microbial pathogens from the canal system using disinfecting protocols during chemomechanical preparation and obturation, minimising cross-contamination from instruments or filling materials [3,4]. Gutta-percha (GP) has been a widely used root canal filling material for over a century, preferred due to its favourable biocompatibility, cost-effectiveness, and extensive clinical application [3]. Even though GP cones are manufactured under aseptic conditions, they can become contaminated during storage [5,6] when handled by aerosols and physical sources [4]. Microorganisms are the primary etiological factor in pulp diseases and apical periodontitis. Therefore, maintaining strict aseptic protocols during dental treatment is essential to minimise microbial contamination and ensure optimal treatment outcomes [7]. The incomplete disinfection of intricate root canal systems and the usage of infected filling materials through cross-contamination increase the risk of persistent bacteria, resulting in endodontic treatment failure [3]. *Enterococcus faecalis* (*E. faecalis*), a frequently isolated species from infected canals [3], has a key impact on obturation material cross-contamination [8] and is closely linked to failed root canal therapy. *E. faecalis* is particularly notable for its role in recurrent periapical periodontitis due to its persistence and resistance to eradication [7]. Dioguardi et al. identified *E. faecalis* as the predominant species associated with persistent root and extraradicular infections [9]. Moreover, other microorganisms are also implicated in endodontic treatment failure [10,11,12], such as yeasts like *Candida albicans* (*C. albicans*) [10,12,13] and *Staphylococcus aureus* (*S. aureus*) that are commonly found on improperly handled GP cones [3]. Du et al., in their study, demonstrated that the coexistence of *C. albicans* and *E. faecalis* enhances the virulence of endodontic biofilms by promoting biofilm formation, increasing tolerance to starvation and alkalinity, and improving biofilm stability against mechanical forces and chemical agents [14]. As a result, periapical inflammation in dually infected root canals is more severe compared to mono-species infections [14]. The use of contaminated GP containing these pathogenic species significantly increases the risk of endodontic treatment failure and contributes to persistent endodontic infections that are difficult to eliminate. *S. aureus* can be found in oral infections such as jaw cysts and orofacial abscesses [15]. The dysbiosis of the mentioned pathogens can be directly related to systemic and metabolic diseases such as diabetes, cardiovascular diseases (infective endocarditis), and periodontitis [15,16]. Thus, GP decontamination is an essential step in endodontic treatment, as it is critical for preventing secondary infections [15]. Various studies have assessed the antimicrobial efficacy of disinfecting solutions for GP decontamination using different microbiological methods, including turbidity measurements [17,18,19] and the confirmation of turbidity results through analyses of colony morphology and Gram staining [4,20,21]. Additionally, other studies employed the disc diffusion test [22,23]. However, the gold standard for quantifying bacteria in microbiological routine diagnostics and research studies remains the quantification of colony-forming units (CFUs) [24]. The quantification of CFUs is a method used to assess the viability of bacterial cells, enabling researchers to evaluate the effectiveness of disinfecting agents in inhibiting bacterial growth by comparing CFU counts between treated bacteria and untreated controls [25,26]. Inconsistencies in antimicrobial testing methods weaken the reliability of conclusions of GP cone decontamination protocols and compromise the formulation of evidence-based future recommendations. Sodium hypochlorite (NaOCl) and chlorhexidine digluconate (CHX) are widely recognised and commonly recommended as irrigating solutions for chemomechanical root canal preparation due to their broad-spectrum antimicrobial efficacy, which is critical for effective bacterial eradication and optimising root canal disinfection [27,28]. Due to their antimicrobial mechanisms, both solutions are extensively used as irrigants for GP cone decontamination [18,29]. NaOCl exhibits broad-spectrum antimicrobial activity against Gram-positive and Gram-negative bacteria by irreversibly oxidising sulfhydryl groups in bacterial enzymes, disrupting membrane integrity, altering metabolism, and causing phospholipid degradation through its chlorine content and high pH [30,31]. While the antimicrobial mechanism of NaOCl involves the destruction of bacterial cells, CHX exerts its bactericidal effect through distinct mechanisms [27]. Higher CHX concentrations induce bactericidal activity by causing cytoplasmic precipitation or coagulation, likely through protein cross-linking, ultimately resulting in cell death [27]. The effectiveness of GP cone decontamination is determined by several factors, including the type of irrigant, its concentration, and the duration of GP cone immersion in the solution [18]. Studies have shown that lower concentrations of NaOCl are effective in disinfecting GP cones against *E. faecalis* with a 10 min immersion time [17], while other findings indicate that neither NaOCl nor CHX can eliminate the pathogen within a 5 min exposure period [32]. Given the limited time available during endodontic procedures, the varying findings on the required disinfection time for GP decontamination highlight the need for guidelines to establish the most time-efficient and effective GP cone disinfection protocol.

Due to insufficient data comparing the efficacy of NaOCl and CHX in disinfecting GP cones at various time intervals and the heterogeneity in antimicrobial efficacy testing methods, further investigation is required to standardise and refine these methodologies. Therefore, this systematic review aims to summarise existing in vitro studies and evaluate the time-dependent disinfectant efficacy of NaOCl and CHX on GP cones, focusing on their effectiveness against microorganisms, such as *E. faecalis*, *S. aureus*, and *C. albicans*, commonly associated with endodontic infections and capable of contaminating GP cones through cross-contamination.

## 2. Materials and Methods

The protocol for this systematic review was based on the Cochrane Handbook for Systematic Reviews of Interventions (Version 6.5) [33]. It was registered on the INPLASY website (DOI: 10.37766/inplasy2025.3.0050) under the registration number INPLASY202530050, accessed on 12 March 2025. The following review complied with the PRISMA statement (Preferred Reporting Items for Systematic Reviews and Meta-Analysis) (Supplementary Materials) [34]. The PICO question (population, intervention, comparison, and outcome) was defined as follows: do GP cones (population) disinfected with NaOCL (intervention) compared to those disinfected with CHX (comparison) demonstrate greater efficacy in decontamination at specific time intervals (outcome)?

### 2.1. Search Strategy

Studies were identified through an electronic search of selected databases (Table 1), with no restrictions on the publication year. The most recent search was conducted on 30 September 2024. Two reviewers (R.A. and E.N.) separately performed searches across six databases: PubMed, Web of Science, Cochrane Library, Scielo, Scopus, and LILACS (Table 1). The search strategy was based on the keywords: ((gutta-percha cones) OR (gutta-percha points) OR (gutta-percha)) AND ((decontamination) OR (disinfection)) AND ((solutions) OR (chemical agents)). The studies that were available in the English language were chosen.

### 2.2. Eligibility Criteria

Study selection followed predetermined inclusion/exclusion criteria (Table 2).

### 2.3. Studies Selection and Data Extraction

The titles and abstracts of the retrieved publications were reviewed to identify their relevance to the aim of the current systematic review. Studies identified as duplicates or irrelevant to the review were excluded from further analysis. A full-text assessment was performed using the inclusion criteria, and articles that met the selection parameters were included in the analysis. Reference lists of the included studies were reviewed. Two investigators (R.A. and E.N.) independently selected studies, data extraction, and the risk of bias assessments in duplicate. In cases of disagreements, a third reviewer (I.D.) was consulted to resolve the issue and reach a consensus. Key characteristics of in vitro studies, including the first author’s name, year of publication, sample size, investigated irrigants, microbial cultures, methodology, method of evaluating antimicrobial efficacy, and results, were extracted from each included study and collected in a standardised Excel file.

### 2.4. Risk of Bias Assessment

Two authors (R.A. and E.N.) independently assessed the risk of bias for each included study by evaluating the following parameters: sample size calculation, randomisation, blinding (of examiner and outcome assessment), control group, standardisation, and antimicrobial efficacy measurement (quantification of colony-forming units (CFUs)). The risk of bias for each included study was evaluated (categorised as high, moderate (some concerns), or low) using the Cochrane risk of bias tool [33] adapted for the current systematic review.

## 3. Results

**Figure 1:**
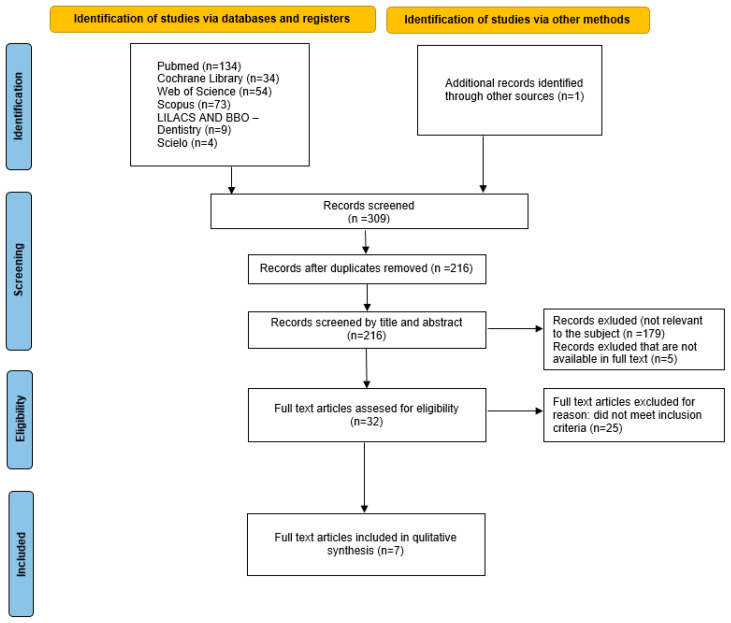
PRISMA flow chart.

A total of 308 records were identified through database searches, and one additional article was obtained from other sources (through manual hand-searching). After 93 duplicate removals, 216 records were reviewed by title and abstract. During the screening process, 179 records were excluded as irrelevant to the subject, and five studies were excluded due to the unavailability of their full texts. The following 32 studies were selected for full-text assessments. After a comprehensive evaluation of the full-text articles, 25 studies were excluded based on methodological limitations. Consequently, seven in vitro studies fulfilled the inclusion criteria and were included in this systematic review (Table 3). The PRISMA flow chart illustrates the selection strategy [34] (Figure 1).

### 3.1. Risk of Bias Assessment

**Figure 2:**
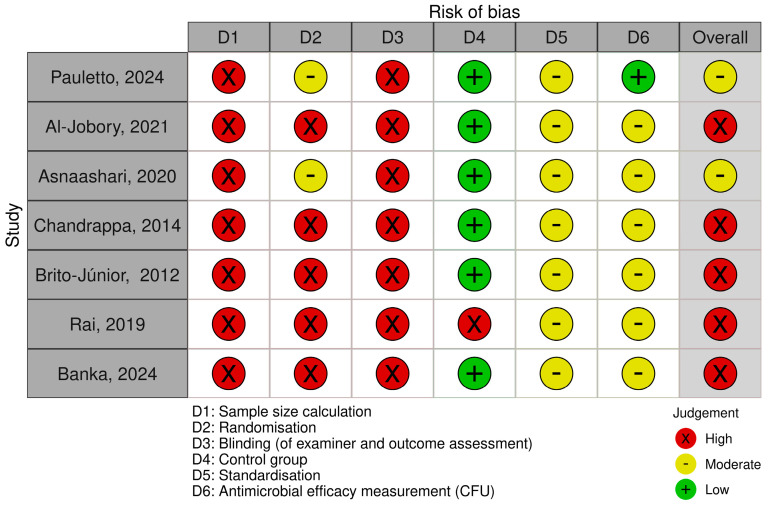
Risk of bias summary [32,35,36,37,38,39,40].

The risk of bias for the seven studies included is summarised in Figure 2 [32,35,36,37,38,39,40]. Five trials were considered to have a high risk of bias, with the most problematic parameters being sample size calculation (in seven studies) [32,35,36,37,38,39,40], outcome assessment blinding (in seven studies) [32,35,36,37,38,39,40], and randomisation (in five studies) [32,35,37,38,39]. The remaining two studies were assessed as having a moderate risk of bias [36,40].

**Table 3:** Studies characteristics, with data extracted from the included studies.

Nr	Leading Author, Publication Year	Sample Size	Tested Irrigants and Their Exposure Time to Gutta-percha (GP) Cones	Tested Microorganisms	Methodology	Evaluation of Antimicrobial Efficacy	Results
1	Pauletto et al. (2024) [40]	98 GP cones	2.5% NaOCl, 2.5% Ca(OCl)_2_, and 2% CHX for 1 and 5 min.	*C. albicans* (ATCC 2508)	GP cones were opened in aseptic conditions and contaminated with the suspension of *C. albicans*. Each group was immersed in each solution for 1 or 5 min and was treated with different methodologies: without agitation, ultrasonic agitation, or agitation with Easy Clean.	After the exposure time to each irrigating solution (1 or 5 min), the samples were examined for turbidity and evaluated for viable colonies. The MIC, fungicidal concentrations, biofilm destruction/inhibition, CFU, and densitometric analyses were assessed.	The most effective in biofilm destruction was observed using concentrations of NaOCl and Ca(OCl)_2_ twice higher than MIC; the biofilm was reduced by 32% and 35%, respectively (*p* < 0.001). The 2.5% Ca(OCl)_2_ solution was effective at all MIC concentrations (*p* < 0.0001). The results showed that in 1 or 5 min of agitation, treatment with 2.5% NaOCl, 2.5% Ca(OCl)_2_, and 2% CHX showed a significant reduction in microbial colonies compared to the saline solution (*p* < 0.05). In both analyses (CFUs and densitometer reading) and at both application times (1 min and 5 min), the use of CHX without agitation proved to be effective in disinfecting cones contaminated with *C. albicans.*
2	Al-Jobory et al. (2021) [35]	40 GP cones	5.25% NaOCl, 2% CHX, and Listerine mouthwash for 1 min.	*E. faecalis* (ATCC 29212)	Sterilised GP cones were immersed in broth media containing *E. faecalis* for 20 min. Each group was immersed in each solution for 1 min. After immersion, GP cones were rinsed, dried, and incubated in nutrient broth for 24 h and 7 days.	The CFUs for *E. faecalis* growth were then calculated for two time points: immediately after GP disinfection (day 0) and after the 7-day incubation period.	NaOCl completely inhibited bacterial growth at day 0 and day 7. CHX completely inhibited bacterial growth on day 0, but the CFU count of *E. faecalis* increased to three units by day 7. Listerine exhibited the highest bacterial growth on day 0 (CFU = 14), but there was a decrease in CFU on day 7, reaching four units.
3	Asnaashari et al. (2020) [36]	70 GP cones	5.25% NaOCl, 2% CHX, and 10% Deconex^®^ 53 PLUS for 1 min. And low-pressure radiofrequency cold plasma (LRFCP) (30 s or 1 min).	*S. aureus* (ATCC 25923)	Sterilised GP cones were immersed in the *S. aureus* suspension and left for 30 min. Each inoculated sample was immersed in one of the disinfecting agents for 1 min, or LRFCP was applied for 30 s or 1 min.	CFUs were counted for each GP cone, with the microbial detection limit set at three CFUs.	All methods showed significant antibacterial activity in comparison with the positive control group (*p* = 0.05). LRFCP and 5.25% NaOCl were significantly more effective than 2% CHX (*p*-values for 30 s LRFCP, and one-minute LRFCP groups and NaOCl were 0.003, 0.001, and 0.003, respectively. The difference between LRFCP and 5.25% NaOCl was not significant (*p* > 0.05).
4	Chandrappa et al. (2014) [32]	280 GP cones	5.25% NaOCl, 2% CHX, and MTAD for 30 s, 1 min, and 5 min.	*E. faecalis* (ATCC29212) and *S. aureus* (ATCC6538)	After artificial contamination, GP cones from each group were separately immersed in the respective disinfectant solutions for 30 s, 1 min, and 5 min.	Antibacterial efficacy was assessed by immersing GP cones in disinfectants, incubating them in thioglycollate media, and counting CFUs digitally.	The mean bacterial count of *E. faecalis* or *S. aureus* was found to be lower after treating the cones with MTAD when compared with other disinfecting solutions for all time intervals tested. The 5.25% NaOCl solution was found to be the second most effective disinfecting solution, while 2% CHX was the least effective among the solutions tested.
5	Brito-Júnior et al. (2012) [37]	60 GP cones	Rosmarinus officinalis extract, 2% CHX, and 2.5% NaOCL for 5 min.	*E. faecalis* (ATCC 4083)	The cones were transferred directly from the package for sterilisation. After contamination, GP cones were transferred to Eppendorf tubes containing 1.2 mL of one of the disinfecting solutions: 2.5% NaOCl, 2.0% CHX, or Rosmarinus officinalis extract. All disinfection procedures were performed for 5 min.	Initially, the antibacterial activity was verified by using a disc diffusion method. Bacterial growth was verified by counting CFUs. The scores used were as follows: 0 indicates the absence of CFUs, 1 indicates lower than 300 CFUs, and 2indicates higher than 300 CFUs.	The positive control had the highest CFU values. There was no difference between the disinfectant solutions evaluated, while all solutions showed similar values to the negative control.
6	Rai et al. (2019) [38]	180 GP cones	6% NaOCl, berberine chloride, chlorhexidine 2%, and cetrimide 0.2% for 1, 3, and 5 min.	*E. faecalis* (ATCC29212) and *S. aureus* (ATCC 6538)	GP cones were taken from freshly opened boxes, arranged in three groups of 30 each, and then immersed in 20 mL of the microbial suspension for one hour. Cones were then transported to sterile paper pads in Petri dishes and allowed to air dry for 10 min. This is followed by the disinfection of the cones in the NaOCL, berberine, and chlorhexidine solutions for time intervals of 1, 3, and 5 min.	The CFUs were graded. Microbial growth was also confirmed with Gram staining, colony morphology, and with a microbial growth identification kit under the microscope by an experienced microbiologist.	The mean bacterial count of *E. faecalis* or *S. aureus* was lower after treating the cones with 6% NaOCL compared with other disinfecting solutions for all time intervals tested. The CHX–cetrimide combination was found to be the second most effective disinfecting solution while the herbal irrigant berberine was the least effective. The mean bacterial count of *E. faecalis* or *S. aureus* was significantly lower (*p* < 0.001) after treating the cones with 6% NaOCL when compared with other disinfecting solutions for all time intervals.
7	Banka et al. (2024) [39]	60 GP cones	5.25% NaOCL, 2% CHX, and 0.1% octenidine dihydrochloride for 1 min.	*E. faecalis* (ATCC2912)	The GP cones were taken out from sealed packets and added into each test tube containing 20 mL of microbial suspensions of activated *E. faecalis* for 30 min. After artificial contamination, GP cones were immersed in the respective disinfectant solutions for 1 min.	After disinfection for 1 min, GP cones were incubated in thioglycollate, transferred to BHI agar, and then incubated aerobically before CFUs were counted using a digital colony counter.	The least number of colonies was seen for 5.25% NaOCL, while the maximum mean number of colonies was seen for the positive control group. It was observed that NaOCL was significantly more effective (*p* < 0.0001) than other disinfectants against *E. faecalis* at a 1 min time interval followed by 0.1% octenidine dihydrochloride.

GP—gutta-percha; NaOCL—sodium hypochlorite; CHX—chlorhexidine; Ca(OCl)_2_—calcium hypochlorite; *E. faecalis*—*Enterococcus faecalis*; *S. aureus*—*Staphylococcus aureus*; *C. albicans*—*Candida albicans*; CFU—colony-forming unit; MIC—minimum inhibitory concentration; LRFCP—low-pressure radiofrequency cold plasma; MTAD—Mixture of tetracycline isomer, acid, and detergent.

### 3.2. Study Characteristics

The data extracted from the included studies are presented in Table 3. Three out of seven studies focused their antimicrobial evaluations on *E. faecalis* [35,37,39]. In their comparisons, two studies assessed the disinfecting efficacy against *E. faecalis*, including *S. aureus* [32,38]. One study focused solely on the disinfecting potential against *S. aureus* [36]. Another research specifically examined the antifungal efficacy of irrigants against *C. albicans* [40]. The most frequently used disinfecting agent was a 5.25% concentration of NaOCl, as reported in four studies [32,35,36,39]. This was followed by a 2.5% NaOCl concentration [37,40] and a 6% NaOCl solution assessed in one study for GP disinfection [38]. A 2% CHX solution was employed in all the included studies [32,35,36,37,39,40], except for one study, which utilised a 2% CHX solution supplemented with a cetrimide mixture [38]. Additionally, one study incorporated agitation of the irrigants to enhance disinfection efficacy [40]. The exposure time of GP cones to disinfecting solutions ranged from 30 s [32] to 5 min [32,37,38,40], with 1 and 5 min durations being the most commonly employed. The included in vitro studies assessed antimicrobial efficacy by quantifying CFUs. Only two studies reported using digital counting for CFUs [32,39], and the others quantified CFUs manually [35,36,37,38,40].

### 3.3. Main Study Outcomes

The efficacy of the NaOCL and CHX solutions was investigated specifically against *E. faecalis* in three studies [35,37,39]. Two studies demonstrated the superior antimicrobial efficacy of 5.25% NaOCl compared to 2% CHX following a 1 min immersion of GP cones in each solution [35,39]. It was found that 5.25% NaOCl completely inhibited bacterial growth (CFU = 0) immediately and after 7 days of incubation [35]. In contrast, 2% CHX inhibited bacterial growth after a 1 min exposure (CFU = 0), but a slight resurgence occurred after 7 days (CFU = 3) [35]. This finding may align with the study in which inter-group comparisons revealed that 5.25% NaOCl exhibited significantly greater efficacy than 2% CHX against *E. faecalis* at the 1 min interval (*p* < 0.0001) [39]. However, despite the observed differences in antimicrobial efficacy, the Al-Jabory et al. study did not report any statistically significant differences between the NaOCl and CHX solutions [35]. Conversely, the study by Brito-Júnior et al. compared lower concentrations of NaOCL (2.5%) and 2% CHX with a prolonged GP cone immersion time of 5 min, finding no statistically significant difference in CFU reduction between the disinfectant solutions (*p* > 0.05) [37].

Two studies investigated the tested solutions’ antimicrobial efficacy against *E. faecalis* and *S. aureus* [32,38]. In contrast, one study evaluated irrigants’ efficacy only against *S. aureus* [36]. Chandrappa et al. demonstrated that at 30 s and 1 min of GP cone immersion, 5.25% NaOCl showed superior disinfecting properties compared to 2% CHX against *E. faecalis* and *S. aureus*. However, 5.25% NaOCl and 2% CHX eliminated *S. aureus* only after 5 min. While NaOCl achieved a more significant CFU reduction in *E. faecalis* compared to CHX, neither disinfectant could eliminate *E. faecalis* even after 5 min [32]. Their study aligns with the findings by Rai et al., which demonstrated that 6% NaOCl was effective against *E. faecalis* within 3 min, while CHX combined with cetrimide required 5 min to achieve antimicrobial effectiveness (*p* < 0.001). In contrast, the NaOCL and CHX–cetrimide solutions were highly effective against *S. aureus* at all time intervals [38]. Two studies reported that NaOCl and 5.25%, 6%, and 2% CHX, respectively, effectively eliminated *S. aureus* from GP cones after 5 min of disinfection [32,38]. The study that assessed antimicrobial efficacy only against *S. aureus* found that after 1 min of disinfection, 5.25% NaOCl reduced the mean CFU count approximately 8.5 times more than 2% CHX (*p* = 0.003), supporting prior findings that NaOCl showed higher antimicrobial efficiency than CHX within 1 min [36].

One study included in this systematic review investigated the antifungal efficacy of the solutions [40]. The study results demonstrated through CFU counting and densitometric analyses that 1 and 5 min with 2% CHX effectively disinfected cones contaminated with *C. albicans* without agitation. It was indicated that 1 or 5 min of agitation with 2.5% NaOCl and 2% CHX resulted in a significant pathogen reduction compared to the saline solution (*p* < 0.05). The 2.5% NaOCl solution showed significantly better results when agitation was applied in comparison to no agitation at both time intervals. The authors concluded that 2% CHX was the only effective solution without agitation across both analysis methods (CFU counting and densitometric readings) at 1 and 5 min [40].

## 4. Discussion

### 4.1. Summary of Evidence

The main cause of failure in endodontic treatment is the presence and persistence of microorganisms in root canals [41]. Factors contributing to this issue include inadequate permanent restoration, ineffective cleaning and shaping, poor canal filling, and using contaminated materials during the procedure [18,42]. Despite being manufactured under sterile conditions and packaged in sealed units, GP cones can become contaminated during storage due to handling, aerosol exposure, and physical sources [29]. The inability to sterilise GP cones at high temperatures requires chemical agents for GP disinfection [17]. Therefore, this study aimed to evaluate the antimicrobial efficacy and time efficiency of NaOCl and CHX in decontaminating GP cones against prevalent endodontic pathogens.

The current systematic review included studies that tested the antimicrobial effects on one or more microorganisms: *E. faecalis*, *S. aureus*, and *C. albicans*. *E. faecalis* was selected due to its ability to survive for extended periods without nutrients and its strong adaptation to the root canal ecosystem [43]. Additionally, *E. faecalis* is the most commonly isolated bacteria in cases of chronic and persistent post-treatment infections [17,42,43,44,45]. *S. aureus* is the most prevalent contaminant of GP cones during storage and after glove handling, highlighting the necessity of GP disinfection [18,46]. However, *C. albicans* is known for being an opportunistic fungal pathogen and is commonly found in persistent endodontic infections [47,48].

Based on the studies included in this review, the most notable quantitative results in CFU formation are observed in GP decontamination using 5.25% NaOCl. The studies demonstrated that GP cone immersion in 5.25% NaOCl for 1 to 5 min exhibited a superior antimicrobial effect against *E. faecalis* compared to CHX [32,35,36,37,39]. In the study conducted by Rai et al., a 6% NaOCl solution was tested and demonstrated superior antimicrobial effectiveness against *E. faecalis* after 3 min [38]. These findings could be explained by previous studies revealing that the antimicrobial activity of NaOCl is concentration-dependent, with higher concentrations inhibiting bacterial growth more quickly than lower ones [29,49]. Aligning with these findings, another study [18] reported that 1% NaOCl eradicated *E. faecalis* and *C. albicans* in 20 min, while 5.25% achieved the same effect in 45 s [4]. In contrast, it was demonstrated that neither 5.25% NaOCl nor 2% CHX could eliminate *E. faecalis* after 5 min of exposure to the contaminated GP cones [32]. Some studies reported that 5.25% NaOCl exhibits potent antibacterial activity against anaerobic bacteria but is less effective in eradicating facultative anaerobic bacteria [50,51]. The conflicting results on the effect of 5.25% NaOCl on *E. faecalis* may vary across studies due to differences in methodology and assessment protocols. According to the included studies, the antimicrobial efficacy of disinfecting solutions varies not only due to their concentration-dependent nature but also based on the duration of exposure of GP cones to the disinfectant. However, as only three studies investigated different exposure durations [32,36,38], it is challenging to comprehensively assess the efficacy of these solutions across varying time intervals. Variability in GP cone exposure time to the microbial suspension may contribute to conflicting results, as prolonged contamination can enhance bacterial adherence, potentially increasing the difficulty of disinfection. Discrepancies among studies may arise from variations in CFU quantification methods, such as comparing results obtained through digital analysis with those from specialist manual counting, which is more susceptible to human error and may impact the overall result accuracy. In addition, conclusions based on colony morphology or disc diffusion tests may be less accurate than CFU counting. Further controlled and standardised studies are needed to either support or disprove the conclusion regarding the efficacy of 5.25% NaOCl in decontaminating GP cones from *E. faecalis*.

The studies included in the qualitative analysis demonstrated effective antimicrobial activity against *S. aureus* following a 5 min exposure to 5.25% [32] or 6% [38] NaOCl and CHX solutions [32,38]. Redmerski et al. concluded that their study aligns with previous research and state that their findings support using 2% CHX for 5 min as an effective disinfectant solution for GP cones [52]. It was found that adding cetrimide to CHX resulted in the complete elimination of *S. aureus* within 1, 3, or 5 min [38]. The literature has shown that the combination of cetrimide and CHX is known to enhance antibacterial efficacy [53,54]. CHX may serve as a suitable alternative for disinfecting GP cones as it avoids the aggressive deteriorative effects of NaOCl on the elasticity of GP cones [6]. CHX demonstrated promising disinfectant effects against *C. albicans*. One study reported that 2.5% NaOCl was more effective than CHX in biofilm destruction. However, it is notable that CHX was the only solution that showed effectiveness without agitation in both CFU and densitometric analyses at both application times (1 and 5 min) [40]. Consistent with these findings [40], 2% CHX demonstrated superior inhibition zones compared to 2.5% NaOCl [55]. Furthermore, 2% CHX disinfected GP cones contaminated with *C. albicans* in 15 s, whereas 2.5% NaOCl required 10 min to achieve similar efficacy [18]. This difference in antifungal efficacy may be explained by the concentration-dependent nature of NaOCl, where lower concentrations (such as 2.5%) result in reduced antimicrobial activity compared to higher concentrations [56]. It can also be attributed to the mechanism of action of CHX, which demonstrates both fungicidal and fungistatic effects by causing nucleoprotein coagulation and structural modifications to fungal cell walls [57,58]. However, differences in the reported antimicrobial efficacy of 2.5% NaOCl and 2% CHX across studies may result from variations in the selected assessment methods. The agar diffusion test may indicate greater efficacy for CHX due to its diffusion properties. In contrast, microdilution assays and biofilm studies offer a more accurate and clinically relevant evaluation of antimicrobial effects. These results encourage future research to investigate CHX as a potentially effective disinfecting solution for GP decontamination. Further studies should assess its antimicrobial properties, potentially in combination with additives, as GP cross-contamination may involve fungal species.

This is the first systematic review that compares NaOCl and CHX solutions for GP decontamination, specifically against *E. faecalis*, *S. aureus*, and *C. albicans* at 0–10 min intervals. The summarised data could serve as an essential reference, emphasising the significance of decontaminating GP cones before root canal obturation procedures. Given the time-sensitive nature of endodontic procedures, understanding the most effective method for disinfecting GP cones is crucial. Based on the results of this systematic review, higher concentrations of NaOCl remain the gold standard, as they effectively eliminate common microorganisms within the 1–5 min interval. However, CHX (both in its standard form and when modified with certain additives, for example, cetrimide) may show promising results in GP disinfection protocols, particularly in cases where fungal species are prevalent. Although GP decontamination is only one component within the complex endodontic treatment procedure, it is crucial to incorporate it into standard disinfection protocols to effectively eliminate microorganisms. This review could encourage researchers to conduct additional studies using more standardised and comprehensive protocols. It could provide a valuable resource for researchers aiming to expand in vitro studies by exploring a broader range of irrigant solutions and concentrations. It could also encourage investigating more diverse and complex microbial populations rather than focusing on a single species, along with a variety of time intervals. Such studies would offer more detailed insights into the effect of irrigants on GP decontamination.

Overall, eliminating bacteria from the root canal system and using disinfected GP can contribute to the long-term retention of an endodontically treated tooth. Preserving a natural tooth in the oral cavity plays a vital role in enhancing a patient’s motivation for continued dental care and maintaining optimal oral hygiene. Maintaining natural dentition improves overall comfort and reduces the need for costly prosthetic restorations or implant placement, which are often required to restore function and aesthetics following tooth loss.

### 4.2. Limitations

Nevertheless, this systematic review has some limitations. There is heterogeneity in the methodologies of the included studies (variations in the microbial species observed, the range of different concentrations of irrigant solutions tested, the time intervals of exposure to disinfecting agents, and differences in the accuracy of antimicrobial evaluation). Five included studies examined the effects of the solutions on only a single microorganism [35,36,37,39,40], while endodontic microbiota is highly complex and diverse [56]. Comparing studies is challenging due to variations in the pathogenic species analysed across different studies. One of the limitations in evaluating the antimicrobial properties of disinfecting solutions is that their efficacy is often tested against individual pathogens, while in endodontic microbiota, microbial communities interact and enhance each other’s survival under unfavourable conditions. These cooperative survival mechanisms complicate the disinfection process, emphasising the need for more advanced and comprehensive methodological protocols. Additionally, the included studies lack testing of different concentrations of irrigant solutions, which may compromise the understanding of their effectiveness. Regarding the time intervals of exposure to disinfecting solutions, only three studies assessed the antimicrobial effect on extended exposure times ranging from 30 s or 1 min to 5 min [32,38,40]. Although all included studies evaluated antimicrobial effects by counting CFUs, only two studies reported using digital methods [32,39]. The subjective evaluation of CFUs can increase human error rates, compromising results’ reliability. More homogeneous, high-quality in vitro studies that incorporate a broader range of endodontic pathogens, a greater variety of irrigant solution concentrations, and a wider range of time intervals for GP exposure to disinfecting solutions are needed.

## 5. Conclusions

Within the limitations of this systematic review, it can be concluded that NaOCl demonstrates high antimicrobial efficacy against *E. faecalis* and *S. aureus* within 1 to 5 min intervals, while CHX shows promising disinfecting effects against *C. albicans*. NaOCl remains the most effective agent for eliminating endodontic pathogens involved in GP cross-contamination, while CHX with additives shows potential against fungal species. Future studies should investigate a broader range of persistent endodontic pathogens within a single study by considering their coexistence and implementing more standardised in vitro models for improved accuracy. Additionally, future research should explore a wider range of irrigant concentrations and exposure times for GP disinfection. Further analysis is necessary to identify the most clinically optimal and applicable disinfection protocol that effectively minimises GP contamination and reduces the risk of secondary infections. Implementing effective disinfection measures is crucial to minimising the risk of GP contamination. This proactive strategy enhances the success rate of endodontic treatment and improves patients’ outcomes.

## Figures and Tables

**Table 1:** Database and keywords.

Database	Keywords
PubMed https://pubmed.ncbi.nlm.nih.gov/	((gutta-percha cones) OR (gutta-percha points) OR (gutta-percha)) AND ((decontamination) OR (disinfection)) AND ((solutions) OR (chemical agents)); Sort by best match (relevance), Abstract, English.
Web of Science https://www.webofscience.com	((gutta-percha cones) OR (gutta-percha points) OR (gutta-percha)) AND ((decontamination) OR (disinfection)) AND ((solutions) OR (chemical agents)); sort by relevance; search language: English.
Cochrane Library https://www.cochranelibrary.com	Title, Abstract, and Keyword: ((gutta-percha cones) OR (gutta-percha points) OR (gutta-percha)) AND ((decontamination) OR (disinfection)) AND ((solutions) OR (chemical agents)); sort by relevance: search language: English.
Scielo https://www.scielo.org/en/	All indexes: ((gutta-percha cones) OR (gutta-percha points) OR (gutta-percha)) AND ((decontamination) OR (disinfection)) AND ((solutions) OR (chemical agents)); search language: English.
Scopus https://www.scopus.com/	Article title, Abstract, and Keyword: ((gutta-percha cones) OR (gutta-percha points) OR (gutta-percha)) AND ((decontamination) OR (disinfection)) AND ((solutions) OR (chemical agents)); sort by relevance; search language: English.
LILACS (added BBO–Dentistry) https://pesquisa.bvsalud.org/	Title, Abstract, and Subject: ((gutta-percha cones) OR (gutta-percha points) OR (gutta-percha)) AND ((decontamination) OR (disinfection)) AND ((solutions) OR (chemical agents)); sort by best match; search language: English.

**Table 2:** Eligibility criteria used for the study selection.

Inclusion Criteria	Exclusion Criteria
In vitro studies	In vivo studies Clinical case report studies Review articles Editorials Opinion studies Abstracts Letters Commentaries Conference Proceedings
Investigating both disinfecting solutions: NaOCL (1–6%) and CHX gluconate (2%)	Only one or none of these solutions
The exposure time of contaminated GP cones to the disinfecting solutions ranged from 0 to 10 min	Exposure time > 10 min
Decontamination against the most prevalent microorganisms associated with endodontic infections: *E. faecalis* and/or *S. aureus* and/or *C. albicans*	The studies focused on testing microorganisms such as *E. coli*, *B. subtilis*, etc., while excluding *E. faecalis* and/or *S. aureus* and/or *C. albicans*
The antimicrobial efficacy of disinfectant solution assessments based on the quantification of CFUs	The antimicrobial efficacy of disinfectant solution assessments based only on turbidity, modified Kirby Bauer disc diffusion, colony morphology, and Gram staining
Full-text availability in the English language with no limitations on the date of study publication	Studies published in non-English languages and unavailable in full-text version

NaOCL—sodium hypochlorite; CHX—chlorhexidine; *E. faecalis*—*Enterococcus faecalis*; *S. aureus*—*Staphylococcus aureus*; *C. albicans*—*Candida albicans*; *E. coli*—*Escherichia coli*; *B. subtilis*—*Bacillus subtilis*; CFU—colony-forming unit.

## Data Availability

Not applicable.

## Supplementary Materials

The following supporting information can be downloaded at: https://www.mdpi.com/article/10.3390/ma18071539/s1.
