# Supplementary material for: The Antimicrobial Efficacy of Sodium Hypochlorite and Chlorhexidine in Gutta-Percha Cone Decontamination: A Systematic Review
Source: Materials (Basel). 2025 Mar 28;18(7):1539. doi: 10.3390/ma18071539 (PMC11990725; doi:10.3390/ma18071539)
Supplement: Supplementary file 1 [file materials-18-01539-s001.zip › materials-3490466-supplementary/Protocol registration.INPLASY Protocol 7555.pdf]

International Platform of Registered Systematic Review and Meta-analysis Protocols

INPLASY

Antimicrobial efficacy of sodium hypochlorite and chlorhexi-dine on gutta-percha cones decontamination: A systematic review

INPLASY202530050
doi: 10.37766/inplasy2025.3.0050
Received: 12 March 2025
Published: 12 March 2025

Aucinaite, R; Nedzinskiene, E; Peciuliene V; Dumbryte, I.

Corresponding author:
Ruta Aucinaite

ruta.aucinaite@gmail.com

Author Affiliation:
Vilnius University, Faculty of Medicine, Institute of Dentistry.

ADMINISTRATIVE INFORMATION

Support - None.

Review Stage at time of this submission - Completed but not published.

Conflicts of interest - None declared.

INPLASY registration number: INPLASY202530050

Amendments - This protocol was registered with the International Platform of Registered Systematic Review and Meta-Analysis Protocols (INPLASY) on 12 March 2025 and was last updated on 12 March 2025.

INTRODUCTION

Review question / Objective Do GP cones (population) disinfected with sodium hypochlorite (NaOCL) (intervention) compared to those disinfected with chlorhexidine (CHX) (comparison) demonstrate greater efficacy in decontamination at specific time intervals (outcome)?

Rationale The success and longevity of endodontic treatment rely on eliminating bacteria from the root canal system, achieved through effective chemomechanical debridement, proper root canal filling, and adequate restoration [1,2]. Endodontic treatment focuses on eradicating microbial pathogens from the canal system using disinfecting protocols during chemomechanical preparation and obturation, minimising cross-contamination from instruments or filling materials [3,4]. Gutta-percha (GP) has been a widely used root canal filling material for over a century, preferred due to its favourable biocompatibility, cost-effectiveness, and extensive clinical application [3]. Even though GP cones are manufactured under aseptic conditions, they can get contaminated during storage [5,6] while handled by aerosols, and physical sources [4]. Microorganisms are the primary etiological factor in pulp diseases and apical periodontitis. Therefore, maintaining strict aseptic protocols during dental treatment is essential to minimise microbial contamination and ensure optimal treatment outcomes [7]. The incomplete disinfection of intricate root canal system and the usage of infected filling materials through cross-contamination increase the risk of persistent bacteria, resulting in endodontic treatment failure [3]. Enterococcus faecalis (E. faecalis), a frequently isolated species from infected canals [3], has a key impact on obturation material cross-contamination [8] and is closely linked to failed root canal therapy. E. faecalis is particularly notable for its role in recurrent periapical periodontitis due to its persistence and resistance to eradication [7]. Dioguardi et al. identified that E. faecalis as the predominant species associated with persistent root and extraradicular in-fections [9]. Moreover,

Aucinaite et al. INPLASY protocol 202530050. doi:10.37766/inplasy2025.3.0050 Downloaded from https://inplasy.com/inplasy-2025-3-0050/

other microorganisms are also implicated in endodontic treatment failure [10-12]: yeasts like Candida albicans (C. albicans) [10,12,13], Staphylococcus aureus (S. aureus) that is commonly found on improperly handled GP cones [3]. The use of contaminated GP containing these pathogenic species significantly increases the risk of endodontic treatment failure and contributes to persistent endodontic infections that are difficult to eliminate. The dysbiosis of the mentioned pathogens can be directly related to systemic and metabolic diseases such as diabetes, cardiovascular diseases (infective endocarditis), periodontitis [15,16]. Due to this reason, GP decontamination is an essential step in endodontic treatment, critical for preventing secondary infections [15]. Sodium hypochlorite (NaOCl) and chlorhexidine digluconate (CHX) are widely recognised and commonly recommended as irrigating solutions for chemomechanical root canal preparation due to their broad-spectrum antimicrobial efficacy, which is critical for effective bacterial eradication and optimising root canal disinfection [27,28]. Due to their antimicrobial mechanisms, both solutions are extensively used as irrigants for GP cone decontaminating [18,29]. The effectiveness of GP cone decontamination is determined by several factors, including the type of irrigant, its concentration, and the duration of GP cone immersion in the solution [18]. Studies have shown that lower concentrations of NaOCl are effective in disinfecting GP cones against E. faecalis with a 10-minute immersion time [17], while other findings indicate that neither NaOCl nor CHX can eliminate the pathogen within a 5-minute exposure period [32]. Given the limited time available during endodontic procedures, the varying findings on the required disinfection time for GP de-contamination highlight the need for guidelines to establish the most time-efficient and effective GP cone disinfection protocol.

Due to the insufficient data comparing the efficacy of NaOCl and CHX in disinfecting GP cones at various time intervals, and the heterogeneity in antimicrobial efficacy testing methods, further investigation is required to standardise and refine these methodologies. Therefore, this systematic review aims to summarise existing in vitro studies and evaluate the time-dependent disinfectant efficacy of NaOCl and CHX on GP cones, focusing on their effectiveness against microorganisms - E. faecalis, S. aureus, and C. albicans - commonly associated with endodontic infections and capable of contaminating GP cones through cross-contamination.

Condition being studied Gutta-percha cross-contamination refers to the transfer of microorganisms onto or via gutta-percha cones from one surface to another. This can occur because of improper storage, exposure to aerosols, or inadequate handling during or after manufacturing. Using contaminated cones with pathogenic microbes significantly increases the risk of endodontic treatment failure by promoting persistent infections that are difficult to eliminate, and it can even lead to apical periodontitis.

METHODS

Search strategy Studies were identified through an electronic search of selected databases, with no restrictions on the publication year. The most recent search was conducted on the 30th of September, 2024. Two reviewers separately performed searches across six databases: PubMed, Web of Science, Cochrane Library, Scielo, Scopus, and LILACS. The search strategy was based on the keywords: ((gutta-percha cones) OR (gutta-percha points) OR (gutta-percha)) AND ((decontamination) OR (disinfection)) AND ((solutions) OR (chemical agents)). The studies that are available in the English language were chosen.

Participant or population

Inclusion

1. Gutta-percha cones;
2. Investigating both disinfecting solutions: NaOCL (1-6 %) and CHX gluconate (2 %);
3. Decontamination against the most prevalent microorganisms associated with endodontic infections: E. faecalis and/or S. aureus and/or C. albicans;

Exclusion criteria

1. Other filling materials, except for gutta-percha cones;
2. Only one or none of these solutions;
3. The studies focused on testing microorganisms such as E. coli, B. subtilis, etc., while excluding E. faecalis and/or S. aureus and/or C. albicans

Intervention Disinfection of gutta-percha (GP) cones with sodium hypochlorite (NaOCl) solutions (1–6%) for immersion times ranging from 0 to 10 minutes.

Comparator Disinfection of gutta-percha (GP) cones with chlorhexidine digluconate (CHX) solutions (2%) for immersion times ranging from 0 to 10 minutes.

Study designs to be included In vitro studies.

Aucinaite et al. INPLASY protocol 202530050. doi:10.37766/inplasy2025.3.0050 Downloaded from https://inplasy.com/inplasy-2025-3-0050/

Eligibility criteria Inclusion
1. In vitro studies;
2. Investigating both disinfecting solutions: NaOCL (1-6 %) and CHX gluconate (2 %);
3. The exposure time of contaminated GP cones to the disinfecting solutions ranged from 0-10 minutes;
4. Decontamination against the most prevalent microorganisms associated with endodontic infections: E. faecalis and/or S. aureus and/or C. albicans;
5. The antimicrobial efficacy of disinfectant solutions assessment based on quantification of colony-forming units (CFUs);
6. Full-text availability in English language with no limitations on the date of study publication.

Exclusion criteria
1. In vivo studies, Clinical case report studies, Review articles, Editorials, Opinion studies, Abstracts, Letters, Commentaries, Conference Proceedings;
2. Only one or none of these solutions;
3. Exposure time > 10 minutes;
4. The studies focused on testing microorganisms such as E. coli, B. subtilis, etc., while excluding E. faecalis and/or S. aureus and/or C. albicans;
5. The antimicrobial efficacy of disinfectant solutions assessment based only on turbidity, modified Kirby Bauer disk diffusion, colony morphology, and Gram staining;
6. Studies published in non-English language and unavailable in full-text version.

Information sources Searches performed across six databases: PubMed, Web of Science, Cochrane Library, Scielo, Scopus, and LILACS. The search strategy was based on the keywords: ((gutta-percha cones) OR (gutta-percha points) OR (gutta-percha)) AND ((decontamination) OR (disinfection)) AND ((solutions) OR (chemical agents)). The studies that are available in the English language were chosen.

Main outcome(s) NaOCl demonstrates high antimicrobial efficacy against E. faecalis and S. aureus within 1 to 5-minute intervals, while CHX shows promising disinfecting effects against C. albicans. NaOCl remains the most effective agent for eliminating endodontic pathogens involved in GP cross-contamination, while CHX with additives shows potential against fungal species.

Additional outcome(s) None.

Quality assessment / Risk of bias analysis Two independent investigators participated in coding the variables and grading the articles included in the systematic review. Each investigator received a blinded copy of the studies and was instructed to provide an evaluation and overall grade for each study. Any discrepancies in scoring were resolved through discussion until consensus was reached. The assessment criteria included sample size calculation, randomization, blinding (of the examiner and outcome), use of a control group, standardisation of conditions, and outcome measurement. The overall quality of the included studies was categorized as high risk, moderate risk, or low risk based on a maximum achievable score of 12 points: 0–4 points indicated high risk; 5–8 points indicated moderate risk; and 9–12 points indicated low risk.
All quality assessments and scoring were performed to minimise the degree of bias.

Strategy of data synthesis The titles and abstracts of the retrieved publications were reviewed to identify their relevance to the aim of the current systematic review. Studies identified as duplicates or irrelevant to the review were excluded from further analysis. A full-text assessment was performed using the inclusion criteria, and articles that met the selection parameters were included in the analysis. Reference lists of the included studies were reviewed. Two investigators independently selected studies, data extraction, and risk of bias assessment in duplicate. In cases of disagreement, a third reviewer was consulted to resolve the issue and reach a consensus.Three hundred-eight records were identified through database searches, and one additional article was obtained from other sources (through manual hand-searching). After 93 duplicate removals, 216 records were reviewed by title and abstract. During the screening process, 179 records were excluded as irrelevant to the subject, and five studies were excluded due to the unavailability of their full texts. The following 32 studies were selected for full-text assessment. After a comprehensive evaluation of the full-text articles, 25 studies were excluded based on methodological limitations. Consequently, seven in vitro studies fulfilled the inclusion criteria and were included in this systematic review.

Subgroup analysis Not applicable.

Sensitivity analysis We conduct restricting analyses only to specific experimental designs - comparison of antimicrobial efficacy between NaOCL and CHX solutions, the antimicrobial efficacy of disinfectant solutions assessment based on quantification of CFUs, investigating the most common endodontic pathogens: E. faecalis

Aucinaite et al. INPLASY protocol 202530050. doi:10.37766/inplasy2025.3.0050 Downloaded from https://inplasy.com/inplasy-2025-3-0050/

and/or S. aureus and/or C. albicans. We compare these re-analyses to our primary results and discuss any major discrepancies in the final review. If sensitivity analyses indicate substantial variation, we will interpret the results with caution and identify areas for further research.

Language restriction English.

Country(ies) involved Lithuania.

Keywords Disinfecting solutions; irrigants; antibacterial; cross-contamination; Enterococcus fae-calis; Staphylococcus aureus; Candida albicans; obturation materials.

Contributions of each author

Author 1 - Ruta Aucinaite.
Author 2 - Egle Nedzinskiene.
Author 3 - Vytaute Peciuliene.
Author 4 - Irma Dumbryte.

Aucinaite et al. INPLASY protocol 202530050. doi:10.37766/inplasy2025.3.0050 Downloaded from https://inplasy.com/inplasy-2025-3-0050/
